# Opportunistic Mobility Support for Resource Constrained Sensor Devices in Smart Cities

**DOI:** 10.3390/s150305112

**Published:** 2015-03-02

**Authors:** Daniel Granlund, Patrik Holmlund, Christer Åhlund

**Affiliations:** Luleå University of Technology, Skellefteå, SE-93187, Sweden; E-Mails: patrik.holmlund@ltu.se (P.H.); christer.ahlund@ltu.se (C.Å.)

**Keywords:** smart cities, WSN, sensors, EAP-Swift

## Abstract

A multitude of wireless sensor devices and technologies are being developed and deployed in cities all over the world. Sensor applications in city environments may include highly mobile installations that span large areas which necessitates sensor mobility support. This paper presents and validates two mechanisms for supporting sensor mobility between different administrative domains. Firstly, EAP-Swift, an Extensible Authentication Protocol (EAP)-based sensor authentication protocol is proposed that enables light-weight sensor authentication and key generation. Secondly, a mechanism for handoffs between wireless sensor gateways is proposed. We validate both mechanisms in a real-life study that was conducted in a smart city environment with several fixed sensors and moving gateways. We conduct similar experiments in an industry-based anechoic Long Term Evolution (LTE) chamber with an ideal radio environment. Further, we validate our results collected from the smart city environment against the results produced under ideal conditions to establish best and real-life case scenarios. Our results clearly validate that our proposed mechanisms can facilitate efficient sensor authentication and handoffs while sensors are roaming in a smart city environment.

## 1. Introduction

In the near future, it is expected that a large number of wireless sensor devices and applications will be developed and deployed in cities on a global scale. Collecting sensor information in the city about local conditions ranging from air quality [1] and road surface conditions [2] to the status of garbage containers, are examples of applications for smart city management [3]. There may be variations in terms of access to a permanent power supply and whether the sensor has access to a fixed internet connection. Furthermore, since different sensor types produce different data volumes, there is a need for a multitude of different sensor and radio communications technologies to interoperate.

The traditional Wireless Sensor Network (WSN) consists of a set of fixed wireless sensor devices that communicate with a fixed central gateway [4]. Since the application areas for WSNs are expanding from traditional industrial applications within a building towards large public areas such as cities [3], sensors will need to support mobility within and between different WSNs [5,6,7]. Using a low-power radio technology with reduced communication range further emphasizes the need for sensor mobility support and opportunistic communication models as in [8]. In traditional sensor mobility solutions, mobility is generally handled within a single administrative domain (AD) where the infrastructure is owned and controlled by a single administrative entity [9]. In a smart city environment where sensors might be mobile within a large geographical area, the sensor mobility scope needs to expand to support mobility between several ADs [9].

The Internet of Things (IoT) is the notion of having objects in our presence with computing, sensing, and communication capabilities [10]. The purpose is to enrich our living with devices that can help us measure, actuate, and also inform us about our surroundings. Such “smart” devices may gather data, and interact with neighbor devices with other devices on the Internet [11]. IoT enabled devices include a broad range of application areas, such as industrial, home automation, and smart cities. An IoT network in a smart city environment is typically heterogeneous with a wide variety of devices supporting different wireless technologies, CPU, and power supply [12]. Interoperability and compatibility between devices in such networks necessitate flexible and lightweight communication protocols [12].

Many solutions exist today that aim to either provide a robust and secure WSN [13,14] or to support highly flexible and mobile installations [4,9]. A solution for robustness and security often places limitations on flexibility and mobility and vice versa [13]. Security solutions and sensor authentication protocols in particular, are typically designed to operate within a single AD, and often in a single WSN. In a sensor mobility scenario where sensors move between different ADs, the need for a secure sensor authentication scheme becomes even more prominent [9]. Therefore, there is a need to develop mechanisms that support sensor authentication and mobility in smart cities.

The main contributions of this paper are:

- We propose, develop, and validate EAP-Swift, an Extensible Authentication Protocol (EAP)-based, lightweight sensor authentication protocol specifically designed to meet the aforementioned requirements on both security and lightweight implementation;
- We propose and validate the use of a tree-based Authentication, Authorization and Accounting (AAA) infrastructure for the authentication of mobile sensors. To the best of our knowledge, we are the first to apply evaluate this AAA infrastructure in the area of sensor mobility;
- We propose, develop, and validate a handoff mechanism that facilitates handoffs between wireless sensor gateways based on signal strength and network latency; and
- We evaluate EAP-Swift and the handoff mechanism through real-life test cases in a smart city environment and in a Long Term Evolution (LTE) radio environment in an anechoic radio test chamber.

The reminder of this paper is organized as follows: Section 2 provides background and problem formulation. Section 3 discusses related work within the area. Section 4 presents EAP-Swift and a handoff mechanism for sensor mobility between different administrative domains. Section 5 presents the results and finally chapter 6 concludes the paper and presents future work.

## 2. Background and Problem Definition

**Figure 1:**
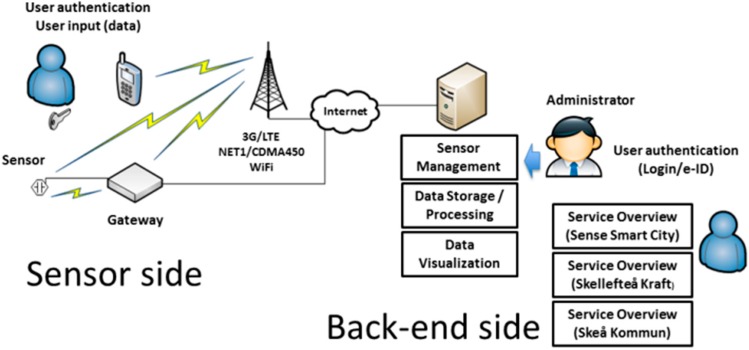
The Sense Smart City Architecture.

Sense Smart City [15] is a Swedish research project that aims to research and develop Informations Communication Technology (ICT) solutions for urban/city environments. As a part of the Sense Smart City project, a sensor platform has been developed based on the Sense Smart City architecture shown in Figure 1. The Sense Smart City architecture consists of a scalable backend server, combined with a modular Application Programming Interface (API) which makes the system suitable to extend with new functionality such as new sensors and applications. The backend system is designed to support a heterogeneous sensor network platform where technologies may co-exist. Since low-power wireless sensor communication technologies typically have limited range, and sensors and/or sensor gateways might be mobile, there is a need for efficient sensor mobility management. Sensor mobility in this context refers to sensor devices’ ability to change connection points between different WSNs and ADs. The traditional WSN design with sensors communicating to a fixed central gateway with internet connection might not be feasible in a Smart City environment that needs to span large areas. By using mobile gateway devices, e.g., deployed in cars or buses, with an uplink internet connection that uses the ubiquitous cellular network, it is possible to have a widespread network of sensors without the need for a fixed infrastructure. Given that the sensor data can be stored in a sensor node if no gateway is within reach, this data can be opportunistically transmitted upon a subsequent reconnection with a gateway. Such opportunistic networking capabilities allows for sensors based on low-power radio technology to effectively reduce power consumption [8].

Traditionally, sensor mobility was limited to a single AD that was owned and managed by the sensor owner; however, problems arise when mobility of sensors is desired where sensors can move between ADs. For example, in order to maintain information security, sensor devices need to be able to authenticate a visited network connection point (gateway). Similarly, the visited network needs to be able to authenticate the visiting sensor before network access is admitted. This functionality would require some pre-established trust relationship between each sensor and each visited network. Since it is not feasible to pre-configure devices with authentication information for every combination of sensor and visited network, there is a need for a more efficient solution.

**Figure 2:**
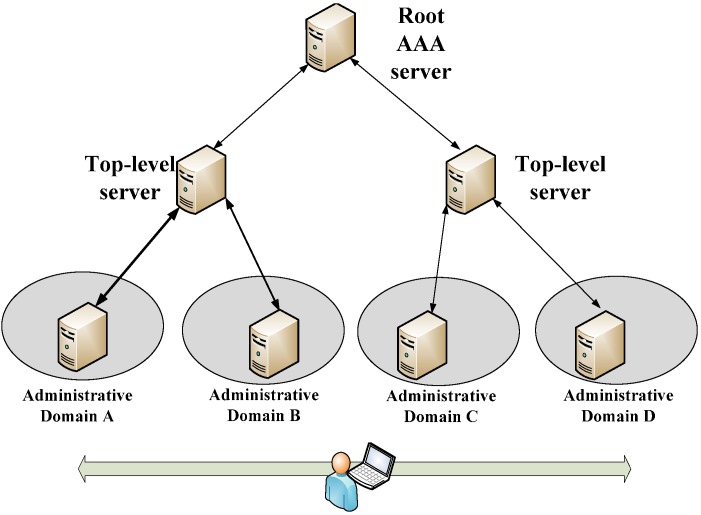
EduRoam tree-like interconnection of AAA servers with different administrative domains.

A parallel can be drawn to a computer network based on WiFi where a user can use their home network authentication credentials to access networks in visited locations. The EduRoam [16] collaboration is an example where this functionality is implemented on a global scale. Academic institutions all over the world are interconnected in a tree-like structure (see Figure 2) using the RADIUS [17] Authentication, Authorization and Accounting (AAA) protocol. This allows users to access WiFi networks at other universities all over the world (e.g., Administrative Domain B, C and D) using their home university (e.g., Administrative Domain A) credentials as long as they are part of the EduRoam collaboration. Each AAA server entity is only required to have a pre-established trust relationship with an overlying node in the tree in order to join the tree. Users are identified using a user@realm style Network Access Identifier (NAI) where the realm is used by the AAA system to route the authentication request to the user’s home AAA server (AAA_H). Authentication is then carried out at the AAA_H and proxied back to the visited networks local AAA server (AAA_L). The AAA_L is then responsible for allowing the user access to the visited WiFi network.

### Problem Definition

The Sense Smart City sensor platform is motivated by the EduRoam architecture, and designed for handling mobility for sensors moving between different ADs. We therefore propose the EAP-Swift protocol for supporting sensor authentication over the RADIUS AAA protocol. A sensor needs to be pre-configured with a sensor ID or NAI along with its authentication credentials in the form of a pre-shared key (psk). Using that information, the sensor will be able to authenticate itself to, and connect to any trusted WSN. As a part of a successful authentication procedure using the EAP-Swift protocol, a session encryption key is generated that can be used to encrypt sensor data on an end-to-end basis. Further, the sensor device should detect and connect to the WSN gateway that offers the best performance at the time, if multiple alternatives exist. Therefore, we propose a handoff mechanism that uses a flexible metric comprising Received Signal Strength_(RSSI), Round-Trip Time (RTT), and jitter for evaluating available WSN gateways.

## 3. Related Work

WSN technologies such as ZigBee [4], Z-Wave [18] and Bluetooth [19] are available in a large number of commercial sensor solutions. A high level of security for wireless transmissions in WSNs can be achieved using encryption protocols based on e.g., elliptic curve cryptography as described in [14]. There are large efforts put into developing solutions for managing sensors in a smart city environment and the research within the IoT-area is comprehensive and well established [12]. IoT solutions targeting smart cities have been proposed [5,6]. Sensor mobility and, in particular, inter-WSN sensor mobility is described [7] as a research area that poses many technical challenges. The aforementioned challenges include heterogeneity, scalability, security, and energy efficiency. In a smart city environment, due to the heterogeneity, large coverage area, and wide range of applications, there will be a need to support sensors moving between different WSNs and ADs [9].

Many mobility solutions exist based on IPv6 over Low Power Wireless Personal Area Network (6LoWPAN) [20] in combination with Mobile IP Version 6 (MIPv6) [21] or Proxy MIPv6 (PMIPv6) [22]. 6LoWPAN based solutions provide IP mobility on a global scale but, due to high signaling costs, are generally not well suited for battery powered applications [23]. A platform for creating large testbeds for wireless sensor applications called WISEBED [24] was developed as a joint collaboration between several European academic institutions. WISEBED supports a large variety of technologies and can be used to simulate heterogeneous WSNs in a smart city environment [25].

As mentioned in the previous section, an EAP-based authentication protocol running on top of a AAA protocol can be used to authenticate sensors roaming between ADs. EAP-based authentication protocols that are intended for use on resource constrained platforms such as sensors include EAP-Sens [26] that provides authentication and session key generation and is based on the EAP-GPSK [27] protocol. EAP-EHash [28] is another protocol that is based on hashing algorithms and provides a high degree of security. Compared to [26,28], this paper proposes EAP-Swift. Like the aforementioned protocols, EAP-Swift is based on hashing algorithms. Key differences include that EAP-Swift is further streamlined in order to fit the needs of our resource constrained sensors. In detail, it relies on two round-trips to the AAA server as compared to three for e.g., EAP-Sens [26]. As compared to EAP-EHash [28], EAP-Swift requires only three hashing operations instead of five hashing- and two 3DES cryptographic operations. This effectively reduces authentication latency and thereby energy consumption for the sensor node which is the main focus of EAP-Swift. Furthermore, it is designed to inter-operate with the RADIUS protocol in an efficient manner.

To support global mobility, EAP-Swift is designed to generate session keys between the sensor and the AAA_H rather than between the sensor and visited network gateway. This enables end-to-end encryption and protection of data in visited networks. Mobility scenarios in smart cities are defined in different ways depending on types of sensors and network (internet) access. Sensor devices may be highly mobile, e.g., wearable sensors that might be attached to a person traveling over large areas [29] or sensors attached to freighted goods that need mobility support [30]. A system is described in [31] which measures air quality in a city environment using vehicle mounted wireless sensors that supports mobility and an opportunistic communication model. Another mobility scenario is to have mobile gateways and sensors in fixed locations [32]. A typical application would be a smart city environment where sensors are placed at road sides and mobile gateways are placed in cars that frequently pass the sensor locations [33]. This design supports the use of low-power sensor nodes with short range radio and opportunistic communication when a gateway node is within range for the wireless connection. Thereby, the system can span a large area without the need for extensive investments in infrastructure.

**Figure 3:**
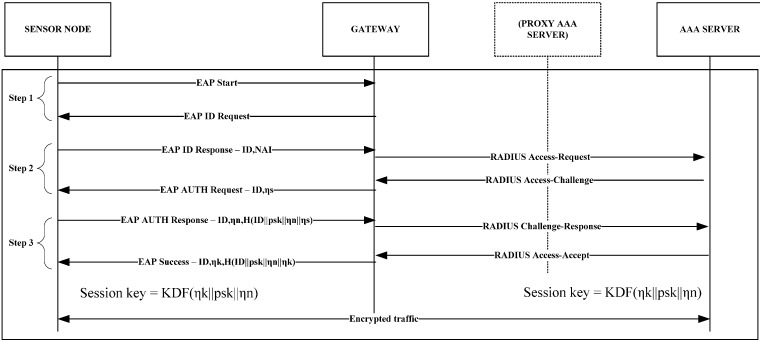
The EAP-Swift protocol authentication and session encryption key generation steps, including message exchange.

In this paper, we present a novel sensor authentication protocol and handoff mechanism that supports mobility between different administrative domains. In particular, compared to the state-of-art in the area of smart cities and sensor mobility, we focus mainly on decreasing the latencies related to sensor authentication and handoff in order to reduce energy consumption for sensor nodes. Compared to handoff mechanisms presented in [22,32,33] that rely only on RSSI for handoff decisions our mechanism considers a more flexible metric comprising RSSI, RTT, and jitter values. This metric is an efficient indicator of the network load in a wireless network and should be taken into consideration for handoff decisions [34]. Our mechanism uses the initial handshake of the proposed EAP-based authentication protocol (Step 1 in Figure 3) to measure the RTT and jitter to the WSN gateway. Upon a handoff decision, the sensor node will already have completed the initial EAP handshake which reduces the overall authentication latency. Further, to the best of our knowledge, we are the first to conduct experimental validation of a smart city sensor mechanism in an industry based anechoic LTE chamber to achieve ideal conditions for evaluation.

## 4. Proposed Sensor Authentication and Mobility Protocol

In this section, we present two mechanisms for enabling secure sensor mobility between different administrative domains (ADs). In particular, we present EAP-Swift for secure sensor authentication. We also present a handoff mechanism to support sensor roaming between wireless sensor gateways in different ADs.

### 4.1. EAP-Swift

To create a sensor authentication protocol that supports the Sense Smart City sensor platform (as described in Section 2), we propose EAP-Swift. EAP-Swift is a hash-based, lightweight authentication protocol, specifically designed to support sensor mobility among different ADs. EAP-Swift uses computationally lightweight hashing algorithms to conserve memory and save battery power by reducing CPU intensive operations such as cryptographic calculations. In particular, upon successful sensor authentication, EAP-Swift generates a session encryption key for the end-to-end encryption of sensor data. Compared to protocols such as EAP-EHash [28] and EAP-Sens [26], EAP-Swift effectively reduces the number of complex calculations as well as reduces the number of round-trips required between the sensor node and the AAA server to complete a full authentication and key generation procedure.

Figure 3 shows a full authentication and session encryption key generation procedure. The procedure begins (Step 1) with the sensor node sending an EAP-Start message. A gateway will then respond with an EAP ID Request. At this stage, the sensor node decides whether or not to proceed with the authentication (useful if many candidate gateways are available). If the sensor node chooses to authenticate to the gateway, an EAP ID Response packet is generated (Step 2) containing the node NAI in the form sensor_id@realm, along with an EAP session identification number (*ID*). It is assumed that, at this stage, the sensor node is pre-configured with an NAI along with a pre-shared key (*psk*). The *psk* is a secret key that is shared only between the sensor node and the home AAA server (AAA_H) and is never transmitted between them. The gateway will then encapsulate the EAP message in a RADIUS message and forward it through the AAA infrastructure to the AAA_H. The AAA_H then generates a 128-bit pseudo random number or, *nonce*, *ns*, which is placed in an EAP Authentication (AUTH) Request packet and is sent back to the sensor node. In the final step (Step 3), the sensor node produces its own nonce, *nn*, which it uses along with the received *ns*, the EAP ID and the *psk* to compute a hash using either MD5 [35] or SHA-1 [36] hashing algorithms.

Since the sensor includes the random *nn* in the hash calculations, it is not possible for a malicious node to trigger the sensor node to produce a hash for a certain input without adding random data. The *nn* along with the computed hash is placed in an EAP AUTH Response message and transmitted to the AAA_H. The AAA_H then has sufficient information to authenticate the sensor node and return an EAP Success message, containing a third nonce *nk*, along with the hash produced by *nk*, *nn*, the EAP ID, and *psk*. Using this information, the sensor is able to authenticate both the AAA_H and to verify the authenticity of the received nonce *nk*. Using *nk* together with the *psk*, both sides are able to produce a session encryption key that may be used for encrypting sensor data. To generate the session encryption key, a Key Derivation Function (KDF) is used which is a simplified version of the Generalized Key Derivation Function, GKDF [27]. The KDF truncates the hash function output to the desired key length. In the simplified version, it is assumed that the desired key length is always shorter than or equal to the length of the produced hash. For the purpose described in this paper, this is a safe assumption since, among the supported hashing functions, MD-5 produces the shortest hash (128 bit) which is equal to the key length supported by the Mulle radio chip [37]. If a longer session encryption key is desired, the GKDF [27] function should be used.

### 4.2. Handoff Mechanism

In the Sense Smart City platform, mobility management is handled by a separate mechanism designed to measure and evaluate the gateways in ADs that offer the best performance at time, *t*. Gateway nodes are configured to periodically broadcast beacons. The sensor node then records the RSSI for every incoming beacon which assists in determining the best connection point (gateway) for connection, if multiple alternatives exist. If the RSSI is higher than a pre-configured threshold value, a presumptuous EAP Start message is issued to the gateway. By recording the time it takes for the message to be acknowledged, the RTT between the sensor and gateway is measured. The RTT values, along with the variation in RTT values, or *jitter*, have proven to be good indicators on the load and congestion of wireless networks [34]. Since RSSI, RTT and jitter are continuously measured and updated (using EAP-Start messages, triggered by every incoming beacon that exceeds the threshold RSSI), a running average value is used to form a metric that can be used for comparison between candidate gateways, see Equations (3)–(5). By tuning the value of *h*, the responsiveness (reaction to rapid changes) of the metric can be adjusted. The running average value of RSSI, RTT, and jitter are then combined together with individual weights (*w_i_*) to form a Policy Value (*PV*) as shown in Equation (6). The weights *W* allow for adjusting the significance of each metric in the composite PV and may be optimized to fit a certain application. Weights are chosen so that $\overset{M}{\underset{m}{\sum }}w_{m}=1$ where w ϵ W and M is the number of metrics used to form the *PV* (in this particular case, 3). The input values are normalized for comparison as shown in Equations (1) and (2). Since a high RSSI value and low RTT and jitter values are preferred, different equations are used for their normalization.

Algorithm 1 describes the gateway selection process in two steps. In the first step (lines 1–6), the set of candidate gateways I is traversed and RSSI, RTT, and jitter are recorded ∀i∈I. In the next step (lines 7–14), a *PV_i_* is computed for all candidate gateways and compared to the *PV* of the currently selected gateway (*PV_icurr_*). If any *PV_i_* > *PV_icurr_*, a handoff to *i* is carried out. If the handoff is carried out successfully, the *PV* of the previously selected gateway is cleared (set to zero) in order to prevent a bouncing (ping-pong) effect. If both the currently active and the candidate gateways are within reach simultaneously, the sensor can initiate the connections and authentication procedures required for attaching to the new gateway before disconnecting the on-going connection. Since a session unique encryption key is negotiated after successful authentication, the sensor needs to change the session encryption key in order to complete the handoff.

To maximize (RSSI):

(1) $$X_{nk}=\{\begin{array}{cc} \begin{array}{c} 1,\\ \frac{X_{nk}-X_{nk_{min}}}{X_{nk_{max}}-X_{nk_{min}}},\\ 0, \end{array} & \begin{array}{l} if\;X_{nk}\geq max(X_{nk})\\ if\;min(X_{nk})<X_{nk}<max(X_{nk})\\ if\;X_{nk}\leq min(X_{nk}) \end{array} \end{array}$$

To minimize (RTT and Jitter):

(2) $$X_{nk}=\{\begin{array}{cc} \begin{array}{c} 0,\\ \frac{X_{nk_{max}}-X_{nk}}{X_{nk_{max}}-X_{nk_{min}}},\\ 1, \end{array} & \begin{array}{l} if\;X_{nk}\geq max(X_{nk})\\ if\;min(X_{nk})<X_{nk}<max(X_{nk})\\ if\;X_{nk}\leq min(X_{nk}) \end{array} \end{array}$$

(3) $${\bar{RSSI}}_{n}=\frac{1}{h}RSSI_{n}+\frac{h-1}{h}{\bar{RSSI}}_{n-1}$$

(4) $${\bar{RTT}}_{n}=\frac{1}{h}RTT_{n}+\frac{h-1}{h}{\bar{RTT}}_{n-1}$$

(5) $${\bar{Jitter}}_{n}=\frac{1}{h}\left|RTT_{n}-{\bar{RTT}}_{n}\right|+\frac{h-1}{h}{\bar{Jitter}}_{n-1}$$

(6) $$PV_{n}=w_{RSSI}{\bar{RSSI}}_{n}+w_{RTT}{\bar{RTT}}_{n}+w_{Jitter}{\bar{Jitter}}_{n}$$

**Algorithm 1** Gateway Selection based on RSSI, RTT, and Jitter**Input:** Candidate gateways, *I***Initialization:**Using *RSSI*, *RTT*, and *Jitter*, discover *I*;1 **foreach** (*i* ϵ *I*) **do**2 | **if**
*RSSI_i_* > *Threshold(RSSI)*
**then**3 | | send EAP-Start to *i*4 | | measure and update RTT/Jitter for *i*5 | **end**6 **end**7 **foreach** (*i* ϵ *I*) **do**8 | compute *PV_i_*9 | **if**
*PV_i_* > *PV_icurr_*
**then**10 | handoff to *i*11 | zero *PV_icurr_* to eliminate ping-pong effect12 | **else**13 | | keep connected to the current gateway *i_curr_*14 **end**

### 4.3. Prototype Implementation

The previous section described a sensor authentication protocol along with a mechanism for supporting sensor mobility and inter-WSN roaming. To evaluate the aforementioned mechanisms, we developed a testbed for experimental evaluation. The sensor and gateway nodes used in the evaluation were based on the Mulle sensor platform [37]. The Mulle sensor node (v6.2) (in this paper, we use the terms Mulle sensor nodes and sensor node interchangeably) consisted of a 16-bit Renesas m16c microcontroller [38], along with a 3 axis accelerometer and an Atmel RF212 868 MHz radio [39]. A small outline (Printed Circuit Board) PCB (24 × 24 × 5 mm) along with components that are optimized for low energy consumption makes the Mulle suitable for applications where spatial and energy consumption requirements are vital. The Mulle microcontroller runs the TinyOS operating system [40]. To carry out the sensor side implementation of the functionality described in the previous section, two TinyOS components were developed; the EAP-Swift based authentication component, and the mobility management (handoff) component.

**Figure 4:**
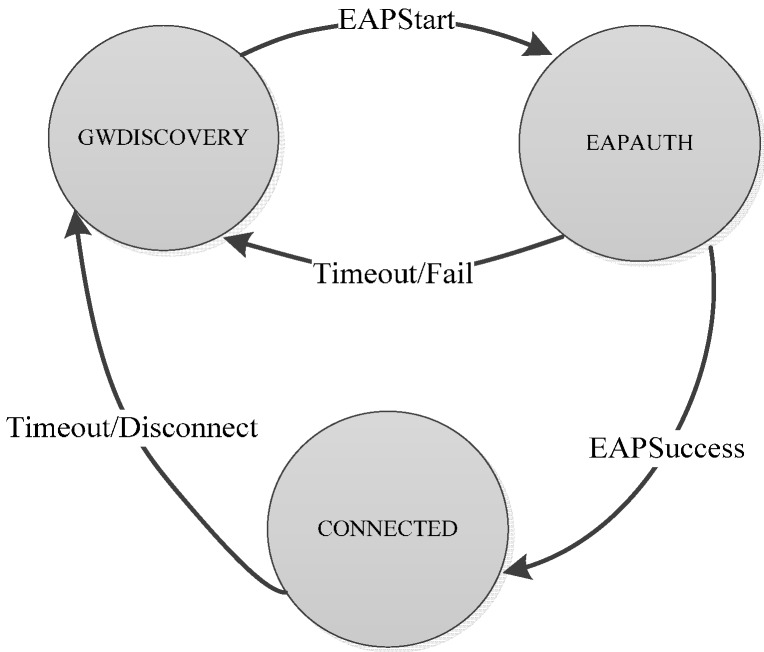
Sensor node connection states and state transitions when connecting to a new sensor gateway.

**Figure 5:**
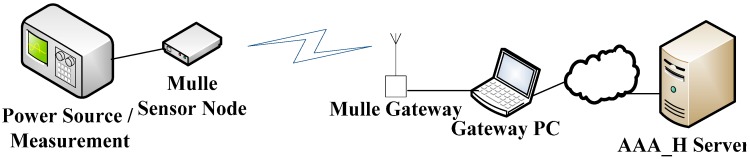
Experimental setup for sensor node power consumption measurement during the authentication procedure.

The EAP-Swift component on the sensor node side consists of an implementation of the protocol, along with a set of C structs to define various messages used for signaling. Implementations of the hashing algorithms were ported to the Mulle platform from open source versions of MD5 [41] and SHA1 [42]. The authentication protocol was implemented as a finite state machine where different states correspond to different stages in the connection process where success/fail/timeout triggers a transition between states as shown in Figure 4. For AAA servers, we considered three 64-bit Ubuntu Linux machines that ran the modular open source RADIUS server software FreeRadius [43]. The EAP-Swift protocol was implemented on the AAA server side as a plugin component in FreeRadius. It should be noted that since the EAP messages are carried within the RADIUS packets, the custom EAP protocol plugin is only needed at the authenticating AAA server (AAA_H) and not in the intermediate proxy AAA servers. The full NesC/C implementation of EAP-Swift and the mobility management component consumes a total of 80 kBytes of code space and 3kBytes of pre-allocated RAM memory on the Mulle platform. Performance evaluation in terms of latency and power consumption for the EAP-Swift authentication mechanism were carried out in a controlled environment using our experimental setup (as shown in Figure 5) that allows for precise time and energy consumption measurements. A Mulle sensor device running the EAP-Swift authentication components was connected to a fixed power supply via a shunt resistor. The shunt resistor allows for precise measurements of the supplied current and voltage using a PicoScope MSO2250 mixed signal digital/analog PC oscilloscope. Furthermore, six digital outputs on the Mulle are connected to digital inputs on the oscilloscope and are programmed to change their value depending on the state of the program execution. This setup enables precise time measurements and detailed analysis of the power consumption for the different stages in the authentication process.

## 5. Results Analysis

This section presents the results related to EAP-Swift and the handoff mechanism proposed in the previous section. Both mechanisms were validated using a prototype implementation and extensive experimentation conducted in a smart city environment and an industry-based LTE chamber to establish the base case and real-life scenarios where sensor mobility needs to be supported. In particular, Section 5.1 presents the evaluation of EAP-Swift with authentication time and power consumption measurements. Section 5.2 presents an experiment where EAP-Swift and the proposed handoff mechanism are validated in a mobility scenario with handoffs between two different wireless sensor gateways. In Section 5.3, an experiment is conducted in a real-life smart city environment as well as in a LTE test chamber. In Section 5.4, a numerical analysis is presented highlighting the effect the protocol performance has on the overall system functionality. Finally, in Section 5.5, a scalability study is performed on the AAA infrastructure using the proposed EAP-Swift protocol.

### 5.1. Evaluation of EAP-Swift

**Figure 6:**
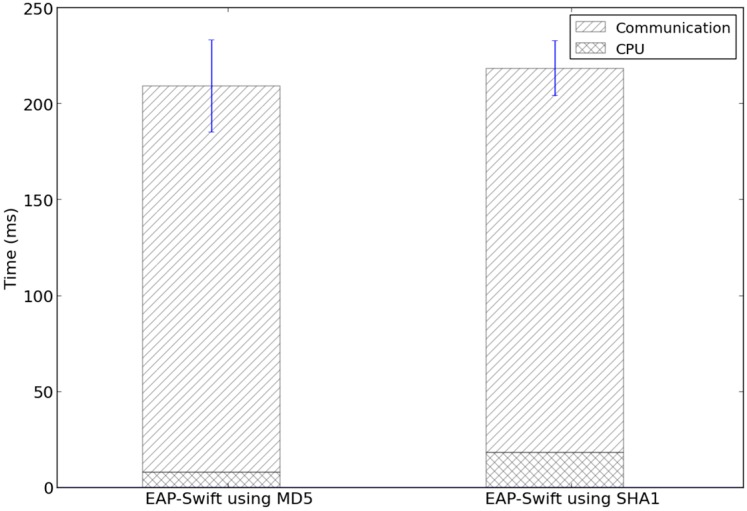
Measured total authentication time for the EAP-Swift implementations based on MD5 and SHA1.

In this subsection, we present results related to the EAP-Swift protocol. To evaluate the EAP-Swift protocol performance we conducted 30 measurements for both MD5 [34] and SHA1 [35] based implementations of EAP-Swift. On the sensor side, time was recorded from the creation of the EAP-ID Response message until the session encryption key was generated after a successful authentication (Step 1–3 in Figure 3). For evaluation, we used the experimental setup as shown in Figure 5 where a Mulle sensor node communicated wirelessly with a gateway consisting of a second Mulle node running a TinyOS BaseStation [44] software that acts as a transparent bridge between the radio and a serial port interface which was connected to a laptop PC running Fedora 14 Linux (Gateway/Authenticator shown in Figure 3). Software in the gateway PC, was developed in Python 2.7 and was responsible for relaying authentication messages to the AAA system. Since this experiment targeted the protocol performance, the AAA system consisted of only one AAA server (running the EAP-Swift plugin). The gateway PC was connected to the AAA server via an Ethernet based network interface. The average RTT between the gateway node and the AAA server was measured to be approximately 2.2 ms with a standard deviation of 1.2 ms. Figure 6 shows the mean authentication time for both versions of the protocol (209 ms for MD5 and 219 ms for SHA1) which was comprised of CPU and communication delay. It can be observed that the communication delay accounts for the majority of the overall authentication latency.

**Figure 7:**
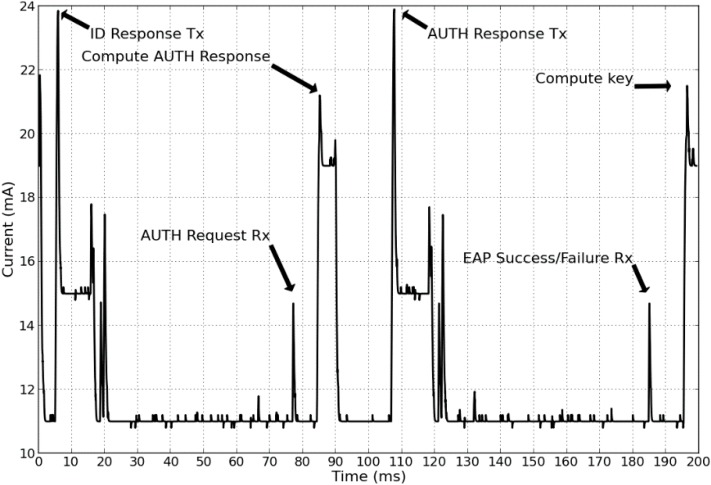
Current consumption by the sensor node during one full authentication procedure using the MD5-based version of EAP-Swift.

This can in part be attributed to the implementation of TinyOS. Figure 7 shows the power consumption for one of the 30 measurements carried out for the MD5 implementation of EAP-Swift. The idle power consumption was 11 mA with an active radio chip. The high peaks at approximately 5 ms and 107 ms correspond to the sending of EAP ID- and AUTH –Response (see Figure 3). Since these were unicast packets, the higher consumption observed immediately after transmission was likely due to the fact that the radio was waiting for a returning acknowledgement. At approx. 78 ms and 185 ms peaks were observed that likely indicate that the replies were received and at 90 ms and 190 ms hashing calculations were performed. Since reception of packets and acknowledgments are carried out on the driver layer in the TinyOS software, we were not able to conclusively verify the exact timing of such events.

The total observed energy consumption during this authentication procedure was approximated using the trapezoidal rule shown in Equation (7) where *N* corresponds to the number of discrete samples. For this experiment, the energy consumed during the authentication procedure starting at time, *a* (see Equation (7)) and ending at time, *b* was approximated based on *N* samples where *f*(*x_k_*) corresponds to the measured power consumption at time = *x_k_*.

(7) $$\int_{a}^{b}f\left(x\right)dx\approx \frac{1}{2}\overset{N}{\underset{k=1}{\sum }}\left(x_{k+1}-x_{k}\right)\left(f\left(x_{k+1}\right)+f(x_{k})\right)$$

For the MD5-based version, the average power consumption of 12.1 mJ was observed with a standard deviation of 0.5 mJ. For the SHA-1 based version, the average consumption of 12.9 mJ with a standard deviation of 0.5 mJ was observed. The results presented in this section validated that EAP-Swift can be used to efficiently support sensor authentication and session key generation using two round-trips to the AAA server. Further, we showed that the communication delay accounts for the major part of the overall authentication delay for both the MD5 and SHA1 based versions of EAP-Swift, which further emphasizes the importance of reducing the communication in the authentication procedure. The next section presents the evaluation of the handoff mechanism.

### 5.2. Evaluation of the Handoff Mechanism

**Figure 8:**
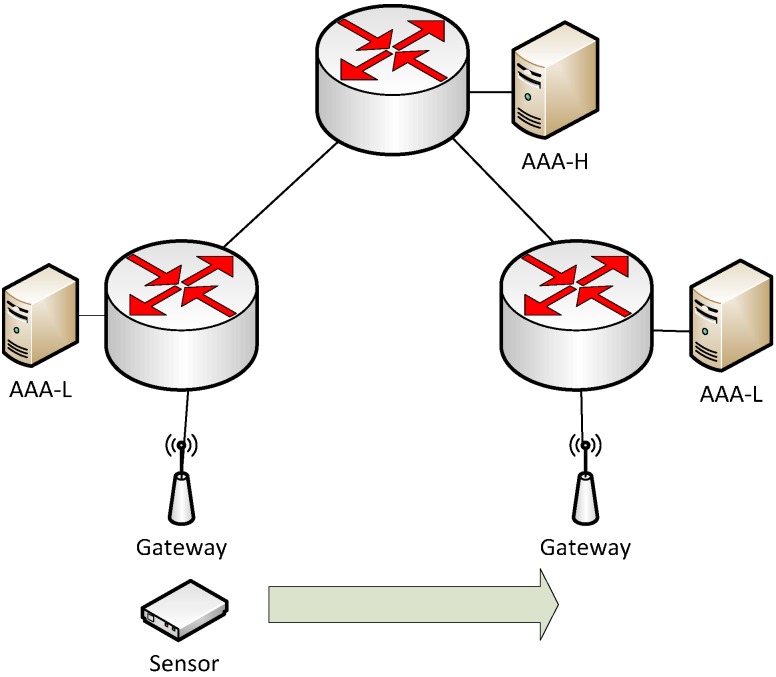
Experimental setup for evaluating the proposed handoff mechanism, with the sensor node roaming between different ADs.

The handoff algorithm was evaluated using a physical setup consisting of two WSN gateway nodes placed 100 m apart in a building corridor (indoors). The gateways were configured to transmit beacons every 500 ms. A mobile Mulle sensor node was moved at walking speed (1.5 m/s) in a fixed straight path between the two sensor gateway nodes. The Mulle sensor was configured to use the MD5-based version of EAP-Swift. To produce sensor data, the Mulle sensor node was configured to sample all three axes of the internal accelerometer at different frequencies to produce different bitrates. The gateways were connected to an AAA infrastructure (shown in Figure 8) with gateways connected to two visited networks (AAA_L). The AAA_L-servers were then connected to an AAA_H being the home AAA server for the sensor. The AAA entities were positioned within different IP subnets and interconnected with 100 Mb/s Ethernet lines. For the handoff mechanism in the sensor node (as described in Section 4) we considered a history window, *h* = 5 (for Equations (3)–(5)). We set equal weights for RSSI, RTT and jitter to compute the final $PV_{i}\;\forall \;i\in I$. Two types of handoffs were evaluated, normal and forced handoff. A forced handoff was triggered by powering off the active gateway, while a normal handoff was triggered using the PVs calculated using Equation (6). As described in Section 4, the forced handoff condition is detected by five consecutive transmission failures. For this experiment, we considered bitrates of 100 bytes/s and 1 kbyte/s. Table 1 shows the packet loss and the apparent application downtime during the handoffs. Our results in this section showed that the sensor data bitrate has a high impact on the packet loss and apparent application downtime during handoffs. In the case of normal handoffs, we observed no packet losses for the 100 bytes/s bitrate. In case of 1 kbyte/s, we observed six packet losses due to the handoff latency. In the case of forced handoffs, the apparent application downtime was higher for the lower bitrate case since the lower packet frequency resulted in the slower detection of lost connection (five consecutive transmission failures). The next section presents the experimental evaluation of EAP-Swift and the handoff mechanism in a smart city environment.

**Table 1:** Handoff mechanism evaluation results for normal and forced handoffs using different bitrates.

	Forced Handoff		Normal Handoff	
	Packet Loss	Application Down Time	Packet Loss	Application Down Time
	(packets)	(ms)	(packets)	(ms)
**100 bytes/s**	5	5000	0	0
**1 kbyte/s**	20	2000	6	600

### 5.3. Evaluation in City Environments

The next step in the experimental verification was carried out in a smart city environment. For this experiment, we considered a different mobility scenario with fixed sensor nodes and mobile gateways. In particular, we deployed battery powered Mulle sensor devices to measure temperature and to provide a car counting functionality based on passive IR sensors. In order to conserve power, the sensor node was configured to turn off the radio and enter a deep sleep mode for 9 s during each 10 s period, rendering in a wake-time of 10%. During the wake period, the sensor was fully functional and listened for incoming beacons from gateway nodes. If connection and data transmission is completed during the wake-time, the sensor will immediately return to the sleep mode rendering in a shorter wake-time (less than 10%). An alkaline 3LR12 4.5 V battery with a capacity of 5400 mAh battery was used to power sensors in the city. Theoretically, this would provide up to a 4800 h battery life for the Mulle sensor using the aforementioned sleep cycle based on power consumption measurements presented in Section 5.1.

**Figure 9:**
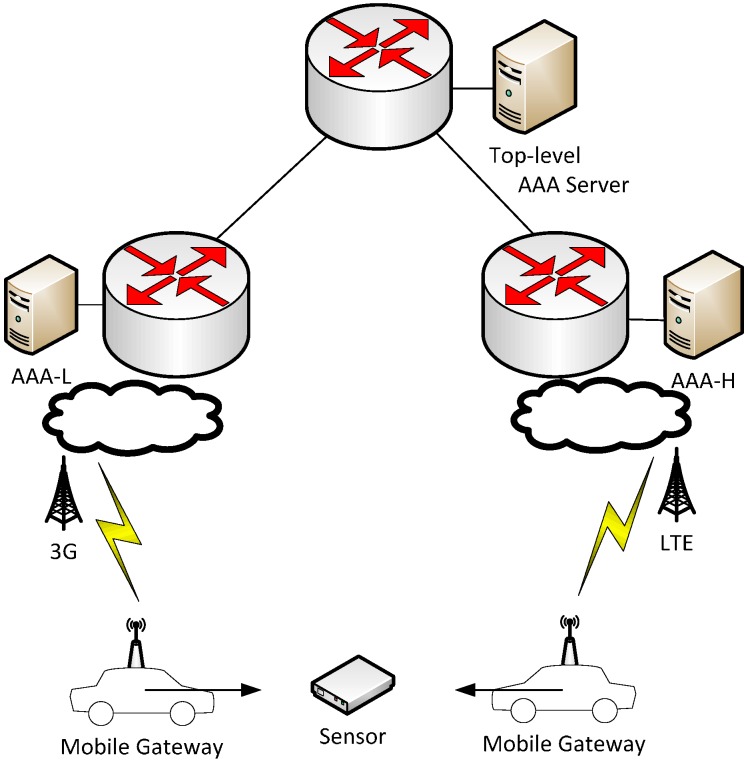
City environment experimental setup with fixed sensor nodes and mobile gateways connected to the cellular network (3G/LTE).

A total of 10 sensor nodes were mounted stationary on light poles and bridges at strategic locations in the city of Skellefteå, Sweden. Gateway functionality was implemented on a Raspberry Pi [45] micro Linux computer connected to the Mulle-based gateway node as described in the previous experiment. Furthermore, a ZTE MF823 LTE modem was used to provide uplink internet access to the gateway. An AAA backend comprised of three interconnected servers where two ADs were formed; AD1 and AD2 with one server for each and a central top level server (see Figure 9). Servers were interconnected using a high speed (1 GB/s, <1 ms delay) ethernet connection. Five sensors were configured with credentials belonging to each AD (at the corresponding AAA_H). The Mulle sensor nodes were configured to use the MD5-based version of EAP-Swift.

The gateway nodes were placed in cars that were likely to pass the sensor locations, the authors were responsible for one gateway each (three in total) that were configured to belong to AD1. The remaining two gateways were configured to belong to AD2 and placed in service cars owned by the local power company which travel frequently within the region. The backend AAA servers were configured to proxy authentication requests to carry out authentication for visiting nodes. Figure 9 depicts the backend configuration of AAA server entities. Since the modem used in this experiment is compatible with LTE, 3G, and EDGE networks, the best available service at each location was automatically selected by the modem from the TeliaSonera public cellular network.

Gateway nodes were configured to transmit beacons every 500 ms. The experiment was carried out during a period of one month. By logging and time-stamping events during sensor authentication, a real-world performance study of the authentication and mobility management protocols was carried out. The total time elapsed from the reception of an EAP-Start message at the gateway until the EAP-Success was returned was recorded in a log file at the gateway. Also, information about whether the sensor was connecting directly to its home AD or to a visiting AD was logged for each successful authentication. Since not all sensor nodes were passed by all mobile gateways during the experiment, two sensor nodes were selected that produced a significant amount of data for analysis. One of the sensors belonged to AD1, and was located in a 3G coverage area and the other sensor belonged to AD2 that was located in a LTE coverage area. Both sensors were frequently passed by the gateways belonging to both AD1 and AD2. In this experiment, the handoff mechanism was used by the sensor node to detect and connect to a gateway in range. Since we never observed any occasion where two or more gateways were within reach from the selected sensors at the same time, no normal handoffs were conducted between gateways during the experiment.

As the next step, the above mentioned real-world scenario was replicated in a lab environment where one sensor node and one gateway were placed in an isolated radio test chamber at Ascom Network Testing [46] that allowed the gateway modem to have exclusive access to a full LTE cell. The Radio Frequency (RF) anechoic test chamber was comprised of a screened room with an attenuation of 115 dB. LTE Transceivers used in the experiment supports two layer Multiple Input, Multiple Output (MIMO), transmission mode 3 and measurements were carried out over a radio link using an Original Equipment Manufacturer (OEM) modem. The purpose of this experiment was to create a best-case scenario and evaluate how the system would perform under ideal conditions. The sensor node, gateway and LTE transmitter antenna were placed at fixed locations inside the test chamber. Since the LTE installation in the test chamber uses Band 4 instead of Band 7 used by TeliaSonera, a Sierra MC7700 modem was used during this experiment. Other than changing the modem, no changes were made to hardware or software as compared to the previous experiment and the same evaluation method was applied.

**Figure 10:**
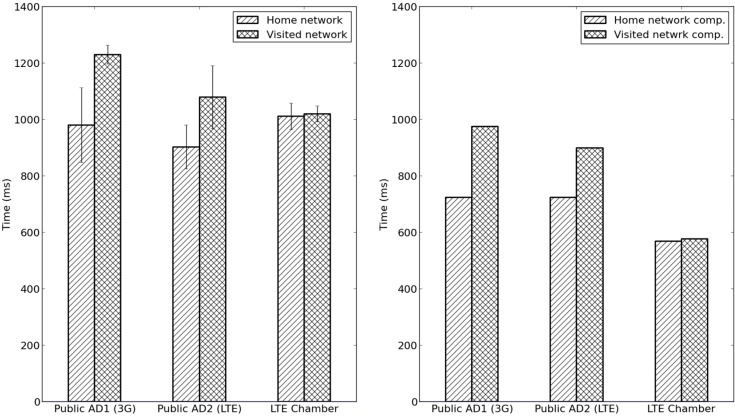
(**a**) Actual measured authentication delay in experiments 3 and 4 in AD1, AD2 and in the controlled radio environment; (**b**) Measured authentication delay as shown in (**a**) with compensation for internet backhaul latency.

Since initially observed authentication delays were significantly higher in our experiments (see Figure 10a) than in the experiment described in Section 5.1 (see Figure 6), we conclude that the access network and backhaul to the AAA server plays a significant role in the overall authentication latency. Therefore, a set of ping measurements were carried out at each location to determine the actual network latency over the cellular network and the internet backhaul respectively. Table 2 shows the result of a series of ping measurements at all three locations. Measurements were carried out both against the AD’s AAA_H server as well as the cellular network Internet gateway to show how much delay is caused by the access network and by the internet backhaul, respectively. The highest latency in both the access network (approx. 300 ms), as well as to the AAA_H server (approx. 475 ms) was measured in the 3G network (AD1). The LTE network showed consistently lower delay values but with noticeable variations while the controlled lab LTE network (test chamber) provided the lowest delay (approx. 50 ms in the access network and 70 ms to the AAA_H server). Table 2 shows the actual measured average authentication delays during live testing at all three locations. Figure 10b shows values for the total authentication delay where the results are made comparable by compensating for the internet backhaul (based on measurements shown in Table 2). Results show that in the controlled lab environment, the latency was lower and more stable which resulted in lower and more predictable authentication delay. It should be noted here that measurements carried out in the controlled lab environment (LTE test chamber) were done with stationary devices whereas in the real-world experiment, the gateway was moving which also may have an impact on the observed latency. A big portion of the latency can be attributed to the type of wireless network as well as the implementation of TinyOS. From this section, it was concluded that EAP-Swift and the proposed handoff mechanism performed well in a real-life experiment scenario. However, the mobile gateway internet connection (3G/LTE) introduced a significant addition to the overall authentication latency as compared to results from the experiment carried out in Section 5.1. The latency in the internet uplink was significantly reduced in the ideal environment provided in the LTE test chamber. The next section provides a numerical analysis on the impact of protocols on the probability of successful sensor authentication.

**Table 2:** Round-trip times from the gateway to the access network internet gateway and the AAA_H server respectively, measured at selected sensor locations used in the experiment presented in Section 5.3.

	RTT in Access Network		RTT to AAA Server	
	Average RTT	Standard Deviation	Average RTT	Standard Deviation
	(ms)	(ms)	(ms)	(ms)
**AD1 (3G uplink)**	308.6	76.1	435.8	268.7
**AD2 (LTE uplink)**	114.0	82.3	122.9	147.2
**LTE Chamber**	50.2	19.3	72.4	2.9

### 5.4. Numerical Analysis

In a scenario such as the one described Section 5.3, there are several factors that may impact the overall functionality of the system. Besides the authentication and communication latencies, the speed at which the gateway passes the sensor node, the sensor node sleep pattern, as well as the radio communication range may have a significant impact on the successful communication of the sensor data. The probability of successful communication of sensor data can be described with the following formulas:

(8) $$P\left(Detection\right)=1-\frac{t_{sleep}-t_{inrange}}{t_{wake}+t_{sleep}}$$

(9) $$P\left(Comm.\right)=\frac{t_{inrange}-t_{trans}}{t_{inrange}}$$

**Figure 11:**
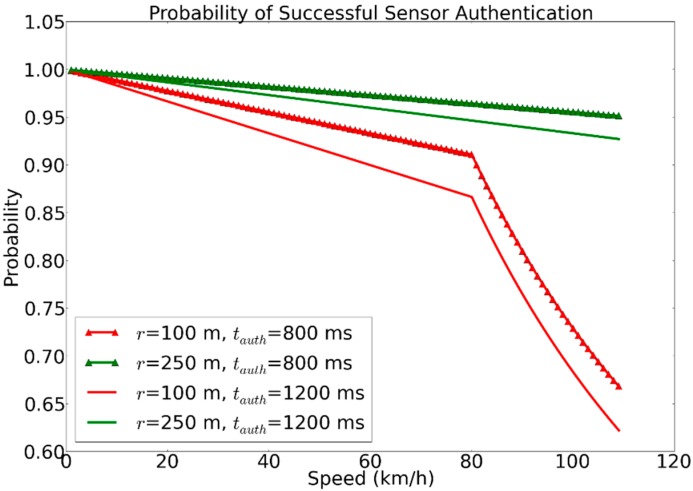
Probability of successful sensor authentication with mobile gateways traveling at higher speeds based on authentication latency.

(10) P(Success)=P(Det.∩ Comm.)

(11) $$t_{inrange}=\frac{2\;x\;r(m)}{1000\;x\frac{v\left(km/h\right)}{3600(s/h)}}=\frac{7.2\;x\;r(m)}{v(km/h)}$$

where *t_sleep_* and *t_wake_* are the sleep and wake-times, *r* represents the wireless communication range, and *v* represents the speed at which the gateway travels. Given that the sensor moves in a linear (straight) path and passes close to the sensor, the time period that the sensor is within range of the gateway, *t_inrange_* can be calculated using Equation (11). The probability of successful data transmission *P*(*Success*) is expressed as the joint probability of successful gateway detection during the wake-time *P*(*Detection*) and successful authentication and communication during *t_inrange_*, *P*(*Comm.*). Figure 11 shows the probability of successful authentication in a scenario with a mobile gateway that passes close to a sensor in a linear trajectory. The plot is based on typical values of 100 and 250 m for maximum wireless communication range, and a fixed sleep pattern where the sensor is awake during 1 s each 10 s period. It can be seen that, with a gateway travelling at a speed of approximately 85 km/h, the probability drops of significantly for the case where *r* = 100 m. The drop can be explained by the higher speed in combination with shorter range introducing the probability that the sensor will never be detected during its wake-time. Plots are shown for two different authentication latencies. Based on average values collected from the experiments presented in Section 5.3, the values 800 and 1200 ms represent typical authentication latencies that can be expected. The value of 800 ms corresponds to using EAP-Swift, and 1200 ms to the state-of-the-art protocols [26,27] that would require three round-trips in order to complete the authentication. In Sweden, the speed limit in a central city area is 50 km/h [47]. Considering a wireless range of 100 m and the aforementioned sleep pattern, the reduction of one round-trip increases the probability of successful authentication from 92% to 94%. The corresponding increase for highway speeds (110 km/h) is 62% to 66%.

### 5.5. Scalability Analysis

To determine the performance and scalability of the FreeRadius server based AAA architecture running EAP-Swift, a benchmark experiment was carried out using the topology presented in Figure 9. In particular, the *radeapclient* tool [43] which is commonly used for RADIUS server performance measurements was modified to support the proposed EAP-Swift protocol. The AAA_H (Intel Xeon 3.1 GHz) was configured with a MySQL-based sensor database (varying in size between 1000–1,000,000 entries) and stress-tests were conducted using the aforementioned tool. Further, the same set of experiments was conducted via the Top-level (proxy) AAA server (Intel Core 2 Duo 3.0 GHz) to determine the proxy capacity of server nodes in the AAA infrastructure. It should be noted that the highest demand on CPU resources is at the authenticating server (AAA_H) and not the root node in the tree. The root node is typically only involved with proxying AAA requests which are simpler tasks (as proven in the experiment). Results from the experiment are detailed in Table 3.

**Table 3:** FreeRadius AAA server performance benchmark running EAP-Swift directly and via proxy (as shown in Figure 9). Maximum number of authentications per second and average CPU utilization.

Entries in Sensor Database	Authentication Carried Out at AAA_H		Authentication Carried Out via Proxy Server	
	Authentications per Second	AAA_H CPU	Authentications per Second	Proxy CPU
**1000**	1218	97%	791	20%
**10,000**	1132	95%	584	15%
**100,000**	1080	94%	581	15%
**1,000,000**	1073	94%	582	14%

The results clearly show that even a moderate server configuration, as the one used in this experiment, can easily handle >1000 authentication requests per second. Given a realistic scenario where a sensor might authentication on an hourly basis, this server would support authenticating >3.6 million sensors. In the case where authentications were carried out via the proxy server, it can be concluded that the network latency (0.8 ms round-trip time on average) between the proxy server and the AAA_H server had a significant impact on the performance. Based on the observed CPU load on the proxy server, it can be determined that the proxy server configuration used would support approximately 3800 proxy requests per second. Further, we observed that between 1000 and 10,000 sensor database entries, there was a substantial reduction in maximum authentications per second whereas above 10,000 entries the reduction was less significant.

### 5.6. Discussion

From the results presented in this section, we conclude that sensor mobility and secure authentication can be supported for battery powered sensor devices using EAP-Swift and the handoff mechanism presented in this paper. From our results, we observed that the communication latency between the sensor gateway and the AAA_H server has a high impact on the overall authentication latency. We highlight that EAP-Swift uses only two round-trips to complete an authentication. Since we did not have access to the implementations of protocols [26,27] we did not make a direct comparison. Rather, we conclude that since [26] proves that at least three round-trips (six messages) are required for the authentication method presented in [27] a significant decrease in energy consumption can be achieved by the reduction of one round-trip as shown by the experiment presented in Section 5.1. Omitting the ciphersuite negotiation phase in [28] will render in the same number of required round-trips for a full authentication procedure as for EAP-Swift. However, the protocol proposed [28] relies on more computationally demanding operations, which have a significant impact on the performance as described in Section 3. Compared to authentication protocols that may require at least three round-trips to complete an authentication procedure and generate an end-to-end key, EAP-Swift reduces the communication latency by 33%.

From the experiment described in Section 5.1 we conclude that approximately 96% of the authentication latency (see Figure 6) is due to the communication of authentication messages exchanged between the sensor node and the AAA_H server. Therefore, using a sensor authentication protocol that requires three round-trips will render in an increase in authentication latency by several hundred milliseconds. The differences in time between authentications carried out in a visited network compared to authentication carried out in the home network indicate that the proxying function in the FreeRadius server has a delay that needs to be taken into consideration (see Figure 11). If a large tree of interconnected AAA servers is formed, the number of intermediate proxy-servers might have a significant impact on the overall latency. The handoff mechanism presented in Section 4 allowed for the timely detection and execution of handoffs between sensor gateways. Packet loss during a handoff can be reduced further by optimizing the scheduling of events (e.g., transmission) in the sensor operating system. The remaining 4% (8.1 ms for the MD-5 version) of authentication latency (shown in Figure 6) is due to CPU processing within the sensor. The Mulle sensor node used in the experiments operated at a clock frequency of 10 MHz. A reduction in clock frequency to save power (e.g., to 1 MHz) would, in this case, result in an increase of the overall authentication latency by approx. 72 ms which would likely be more expensive in terms of battery consumption than the higher clock frequency.

During experimentation, the beacon interval was set to 500 ms. Since the beacon interval directly affects the update frequency for the running average values of RSSI, RTT, and jitter. A smaller interval results in higher resolution in RSSI, RTT and jitter and allows for a smaller value of *h* for the running average value calculations (in Equations (3)–(5)). This can be useful to detect more rapid events, e.g., if the sensor node or gateway travels at a high speed. The increased update frequency, however, will result in increased power consumption on the sensor node. Therefore, the update frequency and the *h* value should be adjusted to fit the application and give the desired responsiveness for the mechanism without excessive resource consumption.

Scalability is an important aspect to consider when building a sensor mobility solution that might include thousands of sensor devices. As validated in Section 5.5, a FreeRadius based AAA infrastructure scales very well, and may handle thousands of authentications per second. In particular, the performance bottleneck in the AAA system depends on, not only the authentication protocol used, but also the authentication delay as well as the number of entries in the user (or sensor) database. If the authentication is carried out using a lightweight authentication protocol such as EAP-Swift, it is likely that the network connection to the AAA server is saturated before the CPU is overloaded [48]. Likewise, if the authentication latency is very high, the server needs to keep RAM memory allocated for the authentication session during a longer time which may cause memory depletion [48]. The aforementioned factors are unlikely with EAP-Swift due to low CPU demand and fast authentications. Focusing on the WSN side, placing a high number of wireless sensor devices with frequent communication in a small geographic area will cause the available WSN bandwidth to be depleted. In such cases, it is desired to keep the sensor authentication overhead to a minimum to maintain efficiency.

As expected, in the smart city environment experiment, we noticed that the sensors placed in an area with LTE network coverage were able to authenticate and transmit data faster compared to sensors in areas with 3G network coverage which resulted in reduced wake-time and thereby, reduced power consumption for the sensor node. We also measured the authentication latency in under ideal conditions by conducting a study in an industrial LTE chamber. Results showed a wide disparity between the best (LTE chamber) and real-life scenarios (public networks). We assert that significant improvements can be achieved using sensor traffic prioritization in the public cellular network without significantly affecting regular data traffic. This way performance and battery life of sensor nodes can be further improved. Depending on the speed at which the gateway passes the sensor, it might be necessary to increase the wake-time or decrease the sleep period, but, for experiments 2 and 3, it was considered sufficient for the sensor to be awake 10% of the time. It is expected that the difference in authentication delay can be larger between the LTE and 3G connected gateways due to the difference in RTT (see Table 2).

## 6. Conclusions and Future Work

This paper proposed, developed and validated EAP-Swift, a novel EAP-based sensor authentication protocol designed to support sensor mobility between different administrative domains. This paper also proposed, developed and validated a sensor mobility mechanism that supports handoff between gateways based on RSSI and RTT in the wireless. The sensor mobility solution presented in this paper shows promising results when it comes to performance and energy efficiency. An important prerequisite for implementing a large number of sensors in a smart city environment is that they are affordable and are easily maintained while providing a robust and functional system. Results presented in this paper confirm that, using an opportunistic communication model, even relatively cheap, battery powered, and resource constrained devices have the ability to form a robust sensor network that can grow on a global scale.

Planned future work includes migrating the tree-like AAA infrastructure to a mobile cloud infrastructure allowing authentication of mobile sensor nodes roaming between different ADs to be carried out closer to the visited sensor network for decreased latency. In particular, we will extend the M^2^C^2^ architecture described in [49] to accommodate the AAA infrastructure. Further, we intend to deploy sensors along with the cloud-based backend in different locations to make a more comprehensive evaluation over time in a larger scale.
